# DHFR Mutants Modulate Their Synchronized Dynamics with the Substrate by Shifting Hydrogen Bond Occupancies

**DOI:** 10.1021/acs.jcim.2c00507

**Published:** 2022-08-19

**Authors:** Ebru Cetin, Ali Rana Atilgan, Canan Atilgan

**Affiliations:** Faculty of Engineering and Natural Sciences, Sabanci University, 34956Istanbul, Turkey

## Abstract

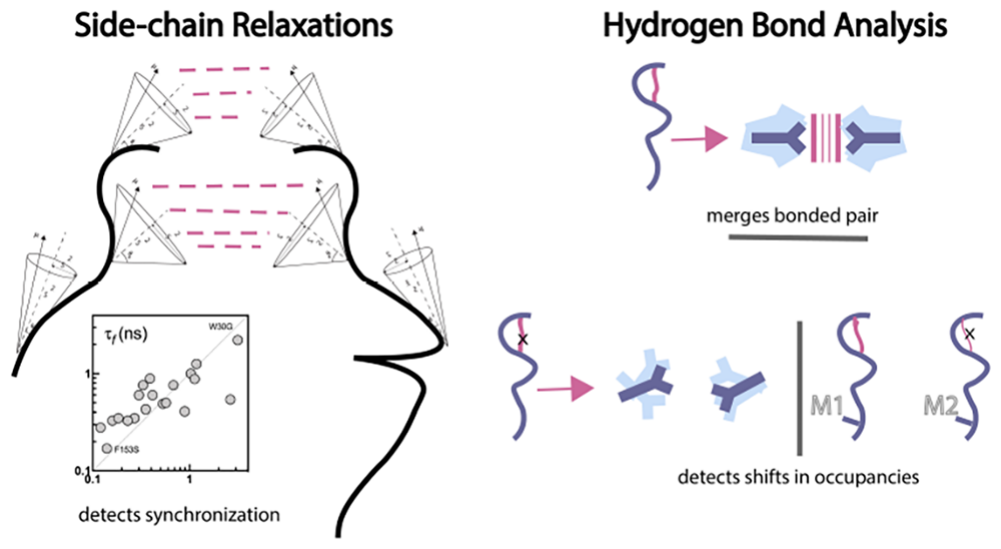

Antibiotic resistance is a global health problem in which mutations occurring in functional proteins render drugs ineffective. The working mechanisms of the arising mutants are seldom apparent; a methodology to decipher these mechanisms systematically would render devising therapies to control the arising mutational pathways possible. Here we utilize C_α_–C_β_ bond vector relaxations obtained from moderate length MD trajectories to determine conduits for functionality of the resistance conferring mutants of *Escherichia coli* dihydrofolate reductase. We find that the whole enzyme is synchronized to the motions of the substrate, irrespective of the mutation introducing gain-of-function or loss-of function. The total coordination of the motions suggests changes in the hydrogen bond dynamics with respect to the wild type as a possible route to determine and classify the mode-of-action of individual mutants. As a result, nine trimethoprim-resistant point mutations arising frequently in evolution experiments are categorized. One group of mutants that display the largest occurrence (L28R, W30G) work directly by modifying the dihydrofolate binding region. Conversely, W30R works indirectly by the formation of the E139–R30 salt bridge which releases energy resulting from tight binding by distorting the binding cavity. A third group (D27E, F153S, I94L) arising as single, resistance invoking mutants in evolution experiment trajectories allosterically and dynamically affects a hydrogen bonding motif formed at residues 59–69–71 which in turn modifies the binding site dynamics. The final group (I5F, A26T, R98P) consists of those mutants that have properties most similar to the wild type; these only appear after one of the other mutants is fixed on the protein structure and therefore display clear epistasis. Thus, we show that the binding event is governed by the entire enzyme dynamics while the binding site residues play gating roles. The adjustments made in the total enzyme in response to point mutations are what make quantifying and pinpointing their effect a hard problem. Here, we show that hydrogen bond dynamics recorded on sub-μs time scales provide the necessary fingerprints to decipher the various mechanisms at play.

## Introduction

Random mutations arising on the protein structure are selected depending on the ambient conditions provided by the organism. In a typical selection experiment, the evolutionary trajectories are monitored under controlled conditions, illuminating the order in which mutations are incorporated to evade the drugs.^1−4^ Furthermore, deep mutational scanning experiments now enable interrogating the range of fitness experienced by the same protein in different backgrounds.^5^ Interestingly, these findings also illuminate trade-offs between activity of a given protein and its stability.^6,7^ In fact, the background not only introduces shifts in the evolutionary landscape but also affects how robustly the landscape is navigated, as shown by directed evolution experiments conducted under strong vs weak selection conditions.^8^ Drug resistance arises amidst these competing mechanisms, since the background fluctuations in a replicating cell are altered in complex ways, providing the conditions for the selection of mutants that would otherwise not be fixed. One strategy to combat severe drug resistance is to guide the pathways to evolutionary dead-ends, by carefully administering drug regimens that do not encourage the rise of robustness enhancing mutations.^9,10^ This approach requires knowledge on the specific mechanisms utilized by the initially arising mutations so that the correct drug derivative may be administered.

The above agenda requires making use of both the kinetics and thermodynamics of the system, which, on the atomistic length scale, requires focusing on the protein where the mutations arise.^11^ While at a given instant a protein is in a specific conformational substate, it in fact samples a range of conformations under physiological conditions. The conformational diversity created with these motions contains enormous numbers of degrees of freedom that will not at all times be coupled to function.^12^ The energy landscape view relates conformational ensembles to their corresponding free energy values.^13−15^ The motions occurring in different energy wells span a wide range of length scales, e.g., from side-chain rotamers, to loop dynamics, to large domain motions. These motions encounter barriers on the rugged energy landscape of the protein, thus presenting functionally relevant time scales from the picosecond all the way to milliseconds and beyond.^16^ While the link between μs–ms motions to function is evident,^17−19^ the contribution of ps–ns motions to the slower time scales remains debated.^20−23^ In the well-known example of adenylate kinase, investigations of backbone dynamics have revealed that the dynamics of hinge regions on the ps–ns time scale are directly connected to enzyme catalysis.^21,24,25^

Mutations introduced on the protein structure further complicate the picture of navigating the conformational space. Most point mutations have no detectable effect on the functioning of a protein compared to the wild type (WT). Others likely directly affect the average three-dimensional structure of the protein and would be detected via various structure determination methods. However, a smaller proportion of mutants do alter fitness without apparent changes; these might act by displaying different dynamics on the fast time scales thus shifting the conformational landscape. To exemplify the range of features to be factored in, consider the example of deep mutational scanning of dihydrofolate reductase (DHFR).^5^ In a follow-up study, the fitness landscape of a DHFR–blue-light sensing LOV2 fusion protein is interrogated to detect allosteric regulation.^26^ The small fraction of function enhancing allosteric residues that exist are sparsely located, displaying enrichment on the protein surface. Such studies reinforce the view that even slight alterations on the energy landscape can improve the fitness *in vivo*, possibly by making use of concerted dynamics.

**Figure 1:**
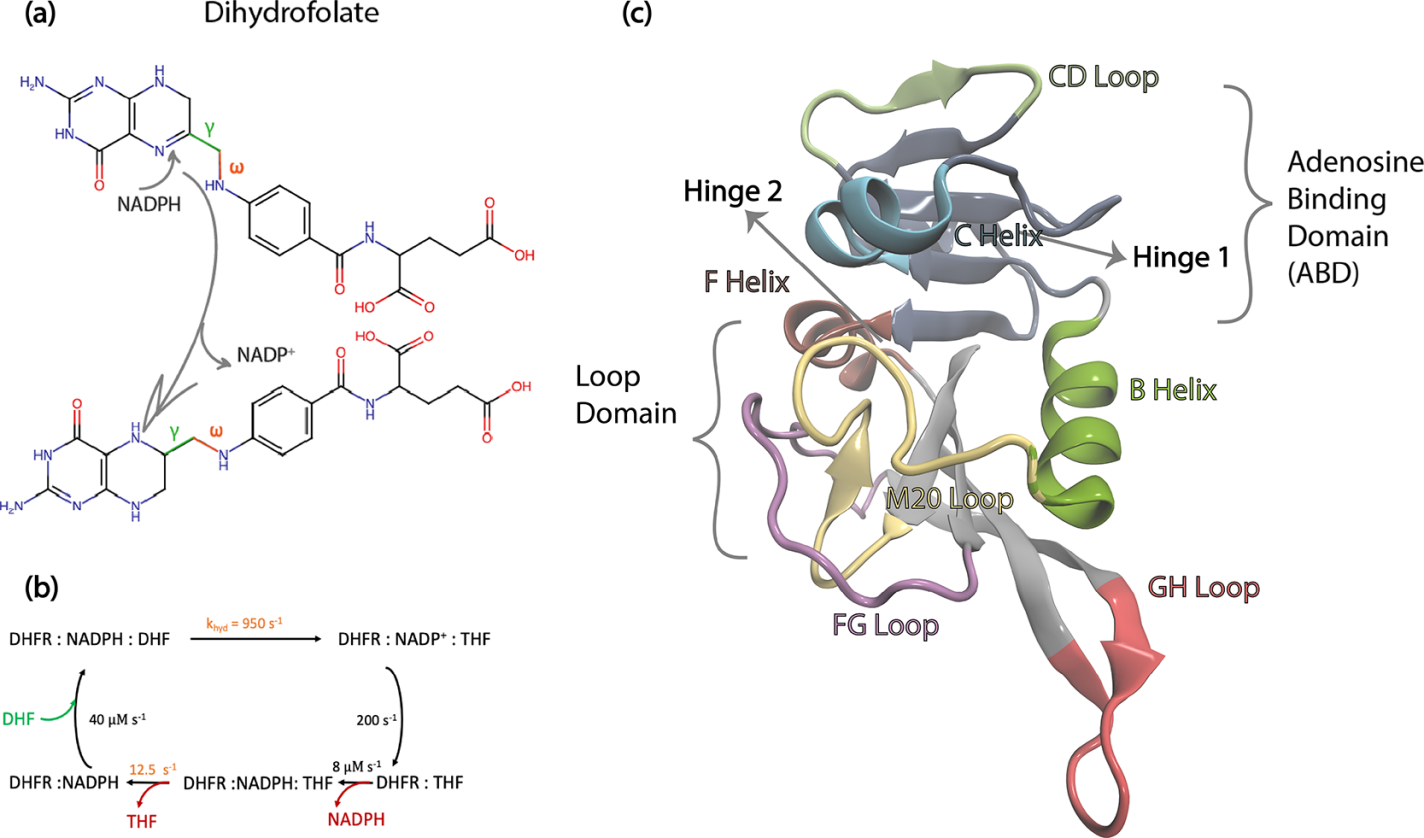
(a) Chemical structure of dihydrofolate; the γ bond is shown in green and the neighboring ω bond in red. The glutamate tail is to the right of the γ bond, and to its left is the pterin ring. Hydride transfer positions indicated by arrows. (b) Catalytic cycle of DHFR. Hydride transfer rate and the rate-limiting step are in orange. (c) DHFR structure (PDB code 1RX2); the colors for the loops and helices are used consistently throughout the paper.

DHFR serves as a model system for the evolution problems at hand.^27−29^ It catalyzes reduction of DHF into tetrahydrofolate (THF), a precursor for purine/thymidylate synthesis (Figure 1a). Loss of THF from the ternary complex (DHFR:THF:NADPH) is the rate-limiting step (Figure 1b).^30^ The structure of the 159 residue long *Escherichia coli* DHFR (Figure 1c) is composed of an α/β arrangement of eight β strands and four α helices. Residues 38–104 make up the so-called adenosine binding domain (ABD), and the rest is the loop domain (LD).^31^ Loops are labeled as the M20 loop (residues 9–24), CD (residues 64–71), FG (residues 116–132), and GH (residues 142–150).^31^ Reduction of DHF occurs through the transfer of two protons. At the hydride transfer step, one proton is supplied by the cofactor NADPH, and the other is extracted from the environment. A significant limitation for studying DHFR is due to the large time scales relevant for enzyme dynamics spanning femtoseconds to seconds.^32^ Interconversion time scales of the reactive substates for ligand binding or catalysis, however, are distributed in the μs–ms range.^33^ Regarding the catalysis mechanism, DHFR experiences multiple conformational changes, including domain rotation and the motion of structural loops. One motion of the structural loops is defined as the occluded state, where the tip of the M20 loop closes over the DHF binding site.^31^

The tiered time scales of protein reactive states have long been studied.^34^ An example is the binding process of lactate dehydrogenase whereby, following the binding of the substrate, the system undergoes a search through many conformations in the μs–ms range, eventually collapsing into a set of states.^35^ In DHFR, ligand binding induces the closed conformation^36^ which constitutes the reactive substates for the hydride transfer. Understanding binding events is essential for determining the underlying biological function, quantified by binding free energy,^37^ whereby the compensation between enthalpy and entropy is utilized.^38,39^ Attempting to optimize one element of the free energy often results in a penalty for the other.^40−42^

In our previous studies, we have determined, by closely examining molecular dynamics (MD) trajectories of the WT and L28R mutant of DHFR, that DHF is dynamically stabilized, thus shifting *k*_cat_ and *K*_m_ to a new optimal.^39^ Therein, hydrogen bonds are dynamically established between several positions on the R28 side chain and DHF, while the average structure is not altered. In fact, the L28R mutant frequently emerges in the presence of TMP,^43,44^ and using this information, we have recently devised a TMP derivative that selectively blocks these dynamical hydrogen bonds and thus obligates the evolutionary trajectories to less detrimental fates.^10^ However, L28R is not the unique resistance conferring mutant that is experimentally observed. Moreover, several frequently encountered mutants never arise in the first step but are strong enhancers of a previously fixed mutation.^43^ We therefore seek to find more automated analyses of MD trajectories to classify the mode of functioning of the point mutations. For this purpose, here we focus on C_α_–C_β_ bond dynamics that have been utilized to study the links between local chain dynamics and global motions in proteins.^45,46^

In this work where we utilize *E. coli* DHFR as our model system, we seek a systematic, unbiased methodology to classify the frequently arising, resistance conferring mutants by analyzing their sub-μs dynamics. The time frame is crucial as several hundred nanosecond-long trajectories provide an optimal for interrogating a series of mutants of a selected protein on computationally feasible times. This length also harbors a rich population of all of the ps–ns time scale events that are postulated to affect the slower motions.^20^

## Methods

### Molecular Dynamics Simulations

All simulations for the systems listed in Table 1 were conducted with the NAMD program.^47^ The water box was set to the dimensions of 65 × 87 × 65 Å with a padding of a 10 Å TIP3P water layer in each direction, making any atom of the protein within at least 20 Å of another atom in its periodic images. Salt concentration is set to isotonic conditions using 0.15 M K and Cl ions. The crystal structure was used in the closed conformation (1RX2) as the initial structure,^31^ and each mutation was introduced with the mutated using VMD Mutator Plugin.^48^ We have utilized the Charmm22 parameter set^49^ with CMAP corrections for proteins, mainly to ensure continuity with our previous work on DHFR whereby the dynamics we sampled with the MD simulations well reproduced the experimental observations. In particular, we have shown that the force field is suitable to differentiate the experimentally determined protonation states of trimethoprim in the D27N and D27S mutants;^39^ we have explained the propensity of select double mutants to confer resistance to DHF,^43^ and we reproduced the experimentally determined X-ray structure of TMP-bound WT and L28R mutant of DHFR.^10^ The systems were minimized for 10 000 steps. Dihydrofolate was simulated in a protonated form; 5-protonated 7,8-dihydrofolate force field parameters were used as reported in the literature.^50^ The particle mesh Ewald sum was utilized to calculate long-range electrostatics with a cutoff distance 12 Å and switching distance of 10 Å. The RATTLE algorithm was applied, and the Verlet algorithm was used with a time step 2 fs. Temperature was controlled by Langevin dynamics with a dampening coefficient of 5 ps^–1^. The pressure was set to 1 atm and regulated by the Langevin piston. The resulting structures were subjected to 210 ns long production runs in the NPT ensemble. In Table 1 we list the systems studied in this work, their average RMSD values, their frequency of observation in morbidostat experiments as the first mutation as well as anywhere in the evolution experiment trajectories, and the average number of hydrogen bonds observed in each trajectory. We have also included some control systems for mutations not observed in the morbidostat; namely, N59A, A107F, D27N, and G121V.

**Table 1:** Systems Studied: Morbidostat Observations, Measured *K*_m_ and *k*_cat_ Values^a^, and Number of Observed Inter-Residue Hydrogen Bonds in MD Simulations

mutant	observed as 1st replacement	1st mutant survived to final genotype	observed in final genotype	*K*_m_ (μM)	*k*_cat_ (s^–1^)	average no. of hydrogen bonds
WT				2.86	5.36	281
I5F	1	0	0	7.68	3.78	263
A26T	0	0	15	7.65	3.70	275
D27E	9	6	8	56.4	14.31	263
L28R	12	9	20	0.95	1.13	257
W30G	4	1	2	9.49	8.18	302
W30R	11	4	9	4.97	8.62	260
I94L	3	2	3	14.87	7.71	274
R98P	2	1	3	34.93	2.82	278
F153S	5	5	9	11.32	5.62	267

a For a total of 28 morbidostat trajectories.^43^

### Hydrogen Bond Analyses

Instantaneous occurrences of hydrogen bonds were obtained by the VMD program Timeline Plugin. The hydrogen bonding criteria were set to 3.0 Å for the distance between donor and acceptor and 20° for the donor–acceptor–hydrogen angle. Multiple hydrogen bonds occurring between any pair of atoms of two residues at a time point are merged into a single occurrence. Thus, the bonds that occur simultaneously contribute to the total duration only once. In this paper, we track those hydrogen bonds on single mutants of DHFR whose occupancies deviate from that of the WT by ±30%. The cutoff is selected as follows: We first made the list of all hydrogen bonds in all the simulated systems, and we made cumulative statistics of the changes in the occupancies in all the single mutants from the WT protein as shown in Figure S1. Most hydrogen bonds have the same statistics across the mutants (within ±5 % of the WT value). Interestingly, this is a symmetric distribution with the losses replaced by a similar number of gains in the number of hydrogen bonds. Moreover, few bonds are outside of the 30% window range indicated by the red dotted vertical lines. Thus, a shift of 30% occupancy (a change that is effective about a third of the time in the dynamics) is a suitable fingerprint for explaining the differences in the mechanisms. This value is also beyond the typical fluctuation of the hydrogen bond occupancies between two independent trajectories of the WT trajectories which has a maximum of 10% change; hence, the choice of 30% also evades the possibility of the error margin contributions due to sampling in the trajectories.

### Model-Free Parameters

Different from our previous work where we have studied the relaxation of the positional fluctuations of *C*_*α*_ atoms in proteins whereby the fluctuation vector is modeled via the first Legendre polynomial,^51,52^ and the fast dynamics is approximated via the Kohlrausch–Williams–Watts expression,^53,54^ here we explore the C_α_–C_β_ bond dynamics. The related bond correlation function is calculated by
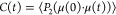
1where μ is the unit vector along the C_α_–C_β_ bond, and *P*_2_ is the second-Legendre polynomial.

The model-free formalism is approximated by a single exponential decay, resulting in a limiting value at long time, *S*^2^, and a characteristic time *τ*_e_.^55^ The model we employ in this work differentiates the contributions of slow and fast motions with characteristic times, *τ*_s_ and *τ*_f_, respectively. Additionally, we assume a distribution of time scales contributing to fast relaxations having a characteristic value, *τ*_f_, rather than *N* known sites with specific relaxation times. With *S*^2^ being the limiting value of the adopted conformation of the bond at long time, *t*
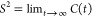
2and a stretched exponent imposed on fast motions,^20^ our model has the form
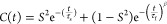
3

The second term in eq 3 represents the summation over many single exponential decay processes^56^ with relaxation times *τ*_*i*_ each having a relative contribution *a*_*i*_ which cannot be resolved individually:
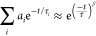
4

**Figure 2:**
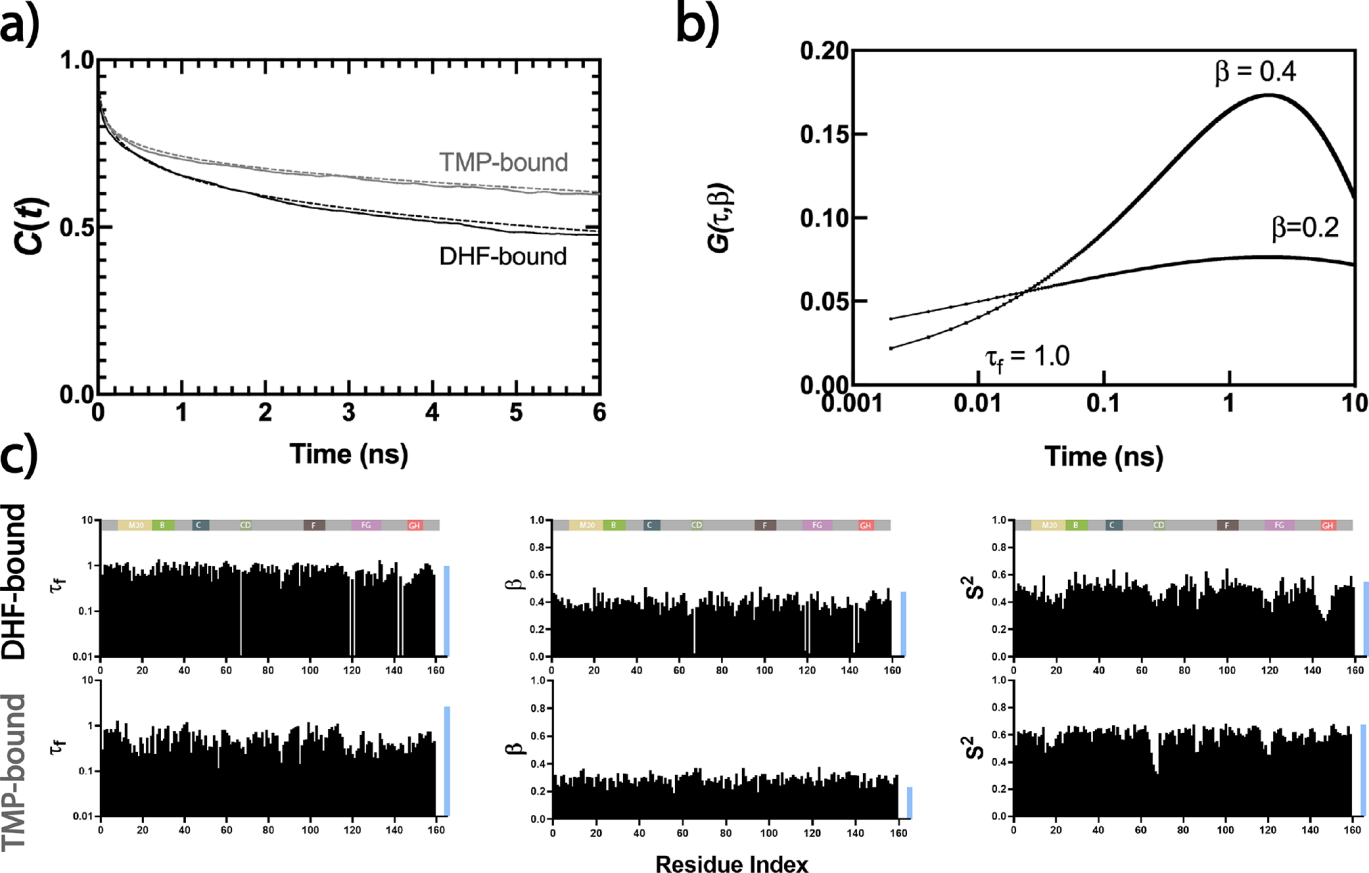
(a) C_α_–C_β_ bond vector relaxations averaged over all residues, displayed for WT DHFR in DHF-bound (black) and TMP-bound (gray) forms; lines fitted by eq 4 are shown by the dashed lines. (b) Distribution of relaxation times for β = 0.4 and β = 0.2; the weighted average *τ*_f_ = 1 ns in both cases. (c) Residue-by-residue curve fit values for fast relaxations in WT DHFR for the DHF-bound and TMP-bound form.

In our procedure, the first 50 ns of each 210 ns long trajectory was discarded as equilibration time. The remaining 160 ns long trajectories, which have converged RMSD values (see Figure S2), were then chopped into 16 chunks of 10 ns length each, as the side chain motions of interest to us have typical time scales in the sub-nanosecond–nanosecond range.^46,100^ For the selection of window size, we have relied on our previous work^20^ that nanosecond time scales impose bounds on the timescale distributions of local dynamics and that relaxation profiles for 7.2 – 24 ns time windows overlap for Cα relaxations; the origins of this behavior are discussed in detail therein. We have indeed tested these window sizes and confirmed that no new relaxation behavior alter the dynamics in this range. Average relaxation functions over all residues were calculated. From the averaged correlation functions, *S*^2^ values were calculated from the 3 to 10 ns portion of the actual correlation function. Then, parameters β, *τ*_f_ and *τ*_s_ were fitted by using global alignment optimization with least-squares. During fitting, the lower bound was selected as 0 for all parameters. The upper bound was constrained to 1 for β, and no constraints were imposed on *τ*_f_ and *τ*_s_. Initial points were chosen as [β = 0.4; *τ*_f_ = 1; *τ*_s_ = 10]. The least-squares curve fit was applied with 50 iterations, and convergence was ensured (see Figure 2a for a sample fit).

In our model, the order parameter *S*^2^ describes the spatial restriction that relaxation behavior adopts when correlation time approaches infinity. Thus, side chains that can explore a larger region of their local environment on the time scales explored will have lower *S*^2^ values. β is a measure of the distribution of the time scales of the many superposed processes governing the motion (eq 4); a high β indicates an energy surface decorated by similar local wells, whereas a low β implicates relaxations spanning several orders of magnitude in relaxation times (Figure 2b). *τ*_f_ is the characteristic time that is due to a wide range of processes influencing the motion of the bond motion in its local minima. We note that *τ*_s_ in these dynamics acts as a fitting parameter that will only provide a qualitative measure of the slower time scale events, since these are partially sampled by our trajectories.

## Results and Discussion

In this work, we propose a mechanical point of view driven by hydrogen bonding and side-chain motions to explain the emergence of resistance in mutants of DHFR. In Table 1 we list the resistance conferring mutants studied in this work, their average RMSD values obtained in the MD simulations, and their frequency of observation in morbidostat experiments (i) as a first mutation, (ii) as their persistence until the end of the experiment once seen as a first mutation, and (iii) as their appearance anywhere in the evolution experiment trajectories.^43^ Measured *K*_m_ and *k*_cat_ values^43^ are also listed along with the total number of hydrogen bonds between pairs of amino acids observed throughout the trajectories. We note that 210 ns trajectories are sufficient to observe the dynamics we wish to probe in this work; for example, the binding free energy calculations carried out on DHFR by introducing various mutations and ligands were shown to reproduce the experimental measurements through the analysis of the last 2 ns of 10 ns long simulations.^9^

Here, we have also analyzed several single mutations selected from Thompson et al. as controls; namely, N59A, A107F, D27N, and G121V. Finally, we have included the double mutants L28R–W30R and L28R–I94L and the triple mutants A26T–L28R–W30G and L28R–W30R–I94L whose resistance conferring mechanisms were elaborated upon at the atomistic scale in our previous work.^43^ The RMSD plots of all systems studied are displayed in Figure S2. We note that we have the TMP-bound counterpart of all of these systems. However, our previous studies have unequivocally shown that the resistance conferring mutants operate via optimizing DHF-bound forms of the enzyme while the TMP-bound forms show minimal conformational and binding affinity changes until at the level of single and double mutants.^39,43^ In this work, we therefore focus our attention on the mutants in DHF-bound states only.

### C_α_–C_β_ Bond Relaxations Differentiate Binding Partner of WT DHFR

Nanosecond time scale motions of individual residues have been known to differentiate epistatic,^57^ collective,^58^ and hence catalytic properties^59^ of proteins. We find that eq 3 provides a good model to describe the dynamics of individual residues on the time scales of a few nanoseconds; the *R*^2^ values of the fits are greater than 0.98 except for less than 1% of the cases fitted. We further find that these relaxations provide a good measure to differentiate the DHFR binding modes of the inhibitor TMP from DHF in its precatalytic complex (Figure 2). Such affinity changes are reflected in the C_α_–C_β_ bond dynamics as (i) a faster initial decay of the correlations as displayed in Figure 2a (average *τ*_f_ shifted from 0.8 ns for DHF to 0.5 ns for TMP binding); (ii) a wider distribution of time scales (i.e., lower β) within the local minima for the inhibitor as revealed by the models in Figure 2b; and (iii) more restricted motions of individual side chains in their local environments in the presence of TMP (*S*^2^ = 0.5 and 0.6 for DHF- and TMP-bound forms, respectively.) Overall, we find that TMP binding rigidifies the protein, and this effect is not limited to the binding pocket but is echoed throughout the protein (Figure 2c).

### Enzyme Side Chain Relaxations Are Synced with Hydride Transfer Bond Motions

When picturing a protein structure, we think of interconverting conformations (substates) at time scales spanning the range from femtoseconds to minutes, changing the spatial arrangement of atoms from bond vibrations to large domain motions.^12^ These substates are realized on fluctuations of the enzyme and distributed as reactive/unreactive and slow/fast on the kinetics. Ligand binding carries the ensemble of states to a narrower set of active conformations. In line with this view, there is experimental evidence that DHFR is found at one major active substate in DHF-bound form.^60^

Structural studies further support that ligand binding assists the stabilization of the native state.^61^ Previous studies have noted a correlation between nanosecond or faster time scale motions in an enzyme to its catalytic activity on ms–s time scales. In fact, protein relaxation and ligand coupling are a well-known phenomenon for myoglobin and heme proteins.^62,63^ Less obvious is a correlation between the local motions such as stretching of a C=O reporting on the catalytic rate^64^ and the sensitivity of a particular bond length to reactivity.^65,66^ Various models have been put forth to explain the bridging of several orders of magnitude time scale differences between, e.g., vibrational time scales of the substrate to the millisecond time scale catalysis rates observed for the enzymes lactate dehydrogenase^12^ and some examples between reactivity and bond length.^65,66^

Since hydride transfer is a rare event, γ-bond relaxations might ensure the enzyme:folate ternary complex scans a large set of coupled states, only a few containing the ones relevant to the turnover. In fact, Falzone et al. found that, reporting on the pterin ring, there is only a single bound form of the DHF, whether in active conformation or inactive conformation.^60^ Additionally, Epstein et al. emphasized that the backbone order parameters of the entire backbone NH relaxations and the relaxations of the binding site are the same.^67^ Here we conjecture that γ-bond relaxations occurring on the same time scales at side chain motions might report on the coupling of enzyme–substrate motions.

**Figure 3:**
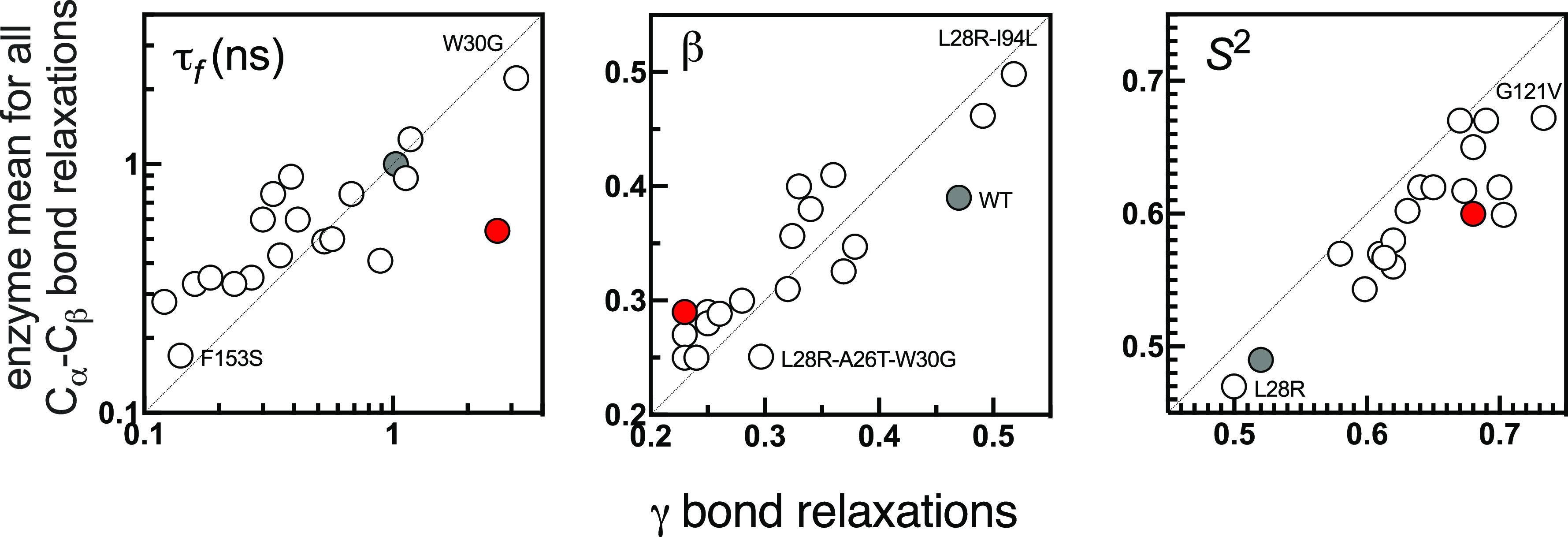
Comparison of enzyme mean (over 159 residues) versus that of γ bond relaxations for all of the systems studied. WT is shown by the gray filled circle. Mutants with extreme values are labeled. *y = x* line shown to guide the eye in each case. Best-fitting lines (not shown) have *R*^2^ values of 0.81, 0.85, and 0.89, respectively, each with *p* value <0.0001. The red dot is the data for the γ bond of WT-TMP showing how the τ_f_ value deviates significantly from that of the enzyme mean, completely destroying the concerted motions.

We indeed find that the side chain relaxations described by our model parameters are extremely well characterized by the relaxations of the γ bond of the substrate (Figure 3) for the whole range of mutants sampled in this work, including the mutants that are deleterious such as A107F, D27N, and G121V. This observation is irrespective of the catalysis rates of the variants and extends beyond the catalytic site residues to the whole protein. For comparison, the counterpart of Figure 3 for only the individual binding cavity residues is shown in Figure S3; in fact, the average over the whole protein gives an improved prediction than any one of the residues directly interacting with the substrate, underscoring the importance of synchronization. Interestingly, the effect is confined to the γ bond, losing its effect to a large extent even for the neighboring ω bond (Figure S4). The general effect of a point mutation is to increase the rate of local relaxations (lower *τ*_f_) while also distributing them over a wider range (lower β); at the same time, the order parameter is mostly upshifted (larger *S*^2^).

In fact, the individual values of these parameters display unique distributions for each mutant (Figure S5). For example, in the WT protein, fast relaxations are relatively similar for all residues and span the 1 ns time scale. On the other hand, mutations disrupt these synced relaxation times. In some cases, the fast time scales are further suppressed (I5F, D27E, F153S) while, in others, they display a wider distribution of times without any significant outliers (e.g., W30R, R98P). In yet others, we observe a few residues that deviate by more than 2σ of the average (e.g., A26T, L28R, and I94L). W30G is particularly slowed down in the local relaxations, on average; it also has many residues whose relaxation times significantly deviate from the average. We note however that these deviating residues cannot be traced to particular regions of the protein, e.g., around the mutated site or in the binding pocket. Furthermore, changes in the local relaxation times by up to 1 order of magnitude do not imply nonfunctionality. In fact, the F153S mutation, which relaxes an order of magnitude faster than the WT while having similar β, is one of the more frequently observed mutations in DHFR (Table 1). Similarly, a broadening in the relaxation times of individual residues cannot be directly related to functionality; e.g., L28R has residues relaxing on sub-nanosecond to 10 ns but is one of the strongest mutations conferred against TMP. Similarly, functionality cannot be pinned on to individual values of the stretch exponent. β for the WT protein is centered on 0.4 as observed for other phenomena in proteins (e.g., C_α_ atom relaxations^20^), and I5F, D27E, and F153S display a similar profile to the WT; however, all others have a smaller β value profile indicating that the processes contributing to the fast relaxations are much disrupted and are distributed to a very wide range of time scales.

Thus, C_α_–C_β_ relaxations report on the small deviations of the energy landscape but are not informative on the origins of the molecular processes at play. Nevertheless, a definitive synchronization of their motions to those of its native substrate using different strategies seems to be operative. Considered with the fact that such an organization does not exist for the tight binding inhibitor TMP (red points in Figure 3), we conclude that the whole DHFR enzyme has evolved to synchronize its side chain motions to the essential movements of its native substrate, particularly to those of the pterin ring. Such a protein-wide synchronization can only be achieved by a network of nonbonded interactions, and we next show that natural fingerprints are provided by hydrogen-bond networks as previously shown for other systems.^68−70^

### Network of Hydrogen Bonds in DHFR is Robust to Point Mutations

We know from previous work that point mutations in enzymes may not affect the average structure in a detectable way, yet their influence on enzyme activity may decide on the fate of the organism.^71,72^ Thus, it is crucial to trace how the dynamics of the protein is changed by mutations to determine the underlying mechanism of action. In previous work, we were able to pinpoint the *modus operandi* of some mutants by detailed analyses of various trajectories of resistance conferring mutations to DHFR, and we have described these unique mechanisms in detail.^39,43^ However, there are other mutants that appear in the morbidostat whose actions do not lend themselves to such analyses. There is accumulating evidence in recent years on how distal regions in a protein utilize various dynamical features to affect the binding site; e.g., thermal conduits that link the protein–water interface to the active site loop in several enzymes provide recent examples that have been well characterized.^73,74^

We thus propose an automated analysis of the occupancies of all hydrogen bonds forming the network of interactions in the folded structure, which, on average, do not display structural changes. The hydrogen bond formation dynamics are influenced not only by slight shifts in the tertiary structure but also by how the vicinal layer of solvent responds to long-range effects of local perturbations.^52^ This provides an excellent opportunity to classify the mechanisms of the single mutants.

We have extracted the hydrogen bonds as described in the Methods section. There is a total of 463 ± 11 hydrogen bonds between pairs of residues in the WT trajectories, and most of these are preserved for the mutants (Table 1). However, we focus on those few whose occupancy changes by ±30% compared to the WT as these imply shifts with dynamical origins that are not reflected in average structures obtained from the very same trajectories (Table 2).

**Table 2:**
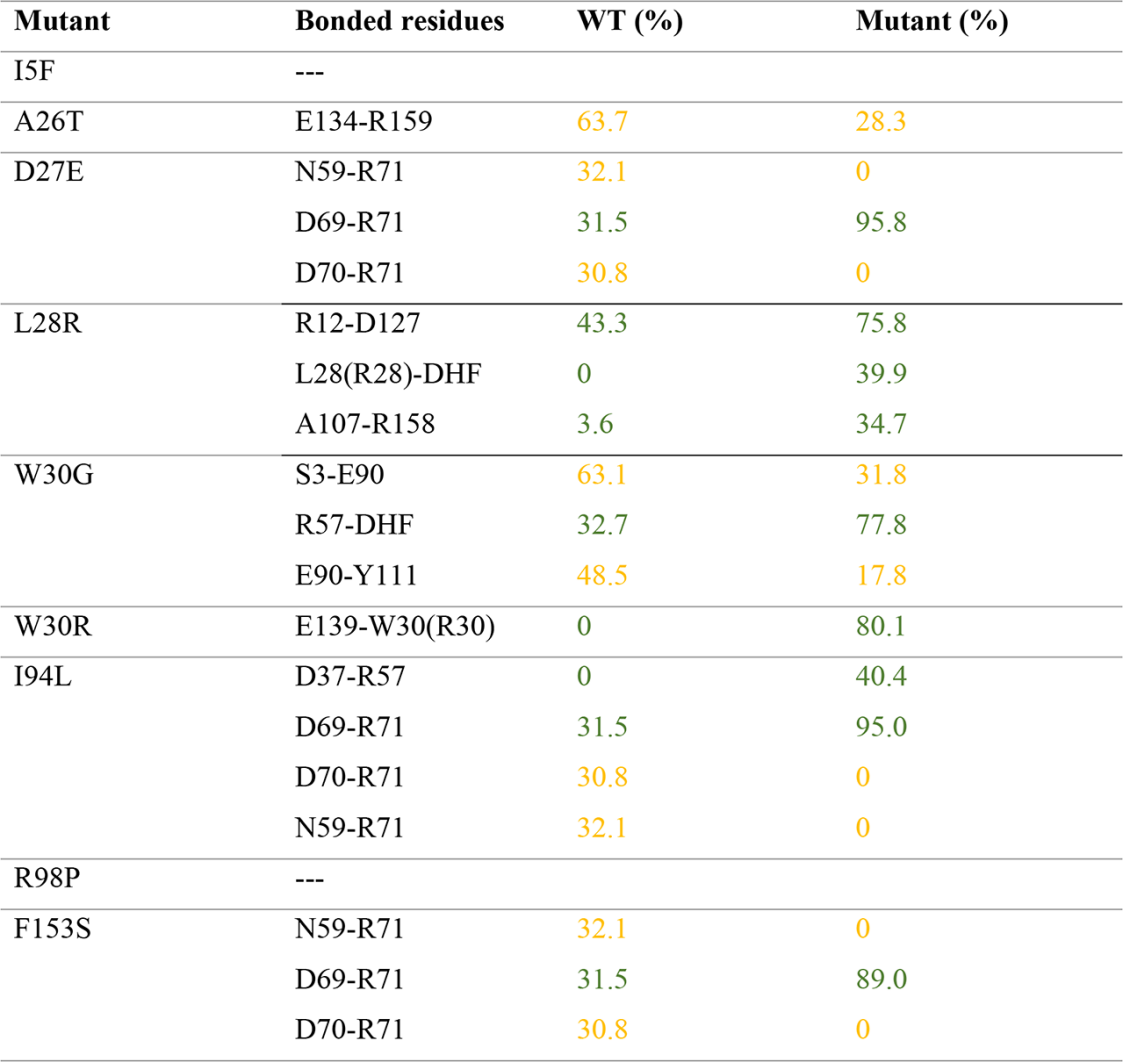
Occupancies of Hydrogen Bonds Specific to the Mutants Extensively Observed in the Morbidostat^a^

a Recorded decrease and increase in occupancies are colored in yellow and green, respectively. Only those for which the difference from the WT value exceeds 30% are listed.

### Hydrogen Bond Dynamics Differentiate Subtle Changes in the Dynamics Due to Point Mutations

Our analysis immediately puts forth the handful of hydrogen bonds whose dynamics are significantly affected by point mutations. Many of the resistance conferring mutants have common changes in their hydrogen bond occupancies, with at least 10% increases for D116–S150 (all), H141–Y151 (all) and D144–N147 (all except W30G). These are accompanied by the disappearance of those in R33–E139 in all cases and over at least 10% decreases in those of R52–DHF (all except W30R). The newly formed bonds (Figure S6) contribute to the immobilization of the GH loop, consistent with previous observations for the WT DHFR, e.g., by an ultraviolet photodissociation mass spectroscopy study confirming the enhanced motions observed in the wild type.^75^ Whether the immobilization of the GH loop is essential for acquiring a mutation requires further investigation. However, here we focus on the more specific changes which are listed in Table 2.

**Figure 4:**
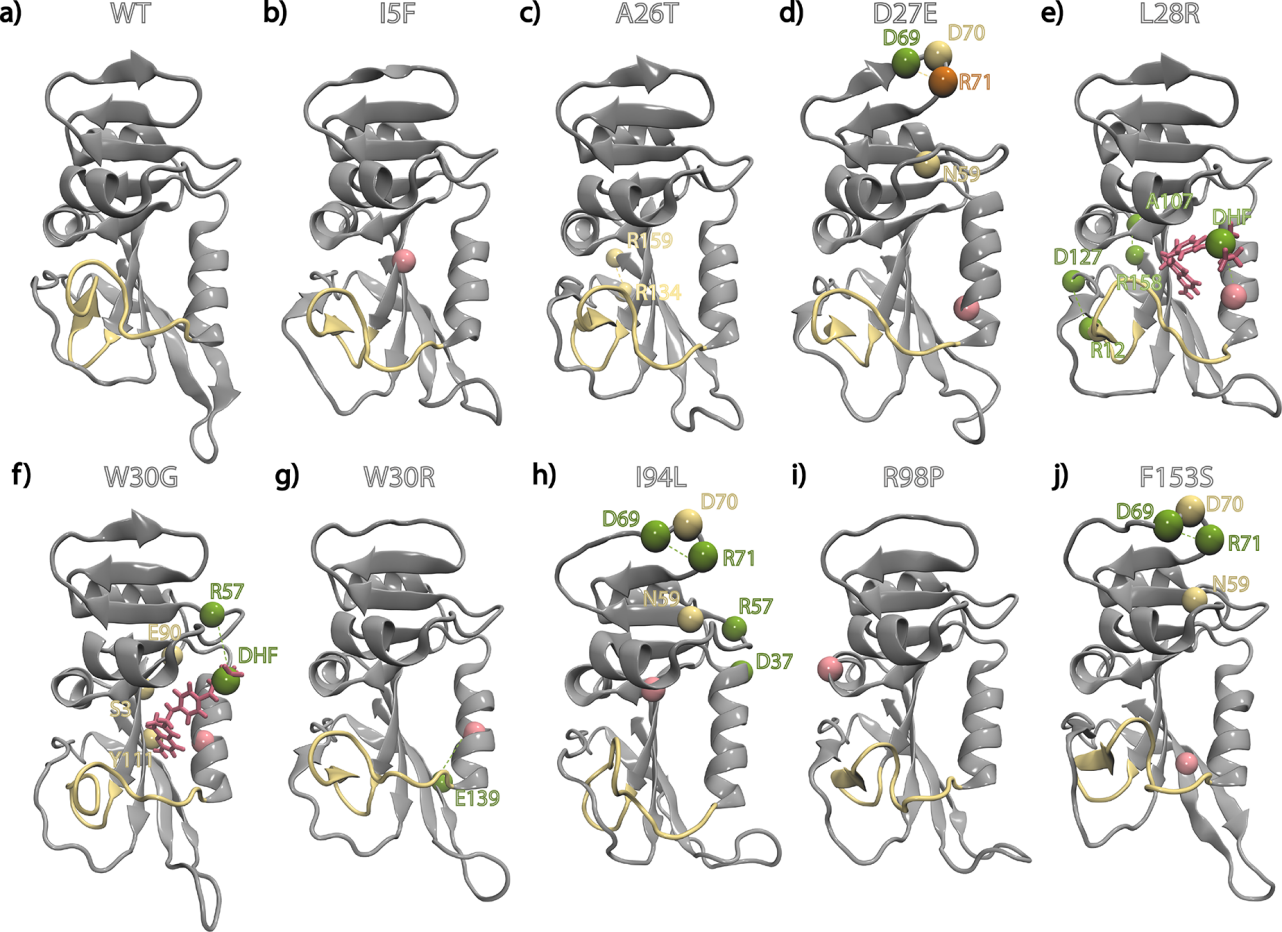
Changes in hydrogen bonding profiles of the mutants with respect to the WT where the M20 loop is displayed in yellow ribbon representation. Residues for which there is significant change in hydrogen bond occupancies are shown as spheres. Rose, mutated residue; green, residue with formed/increased occupancies; yellow, residue with lost/decreased occupancies; tangerine, residue with shifting occupancy from one partner to another.

These are visualized in Figure 4 for the frequently observed single mutants in the morbidostat.^43^ In fact, I5F and R98P, which very rarely appear as a first mutation nor survive to the final genotypes (Table 1), display no such changes (Figure 4b,i). A26T, which does not appear as a first mutation but is frequently observed in the final genotypes and is therefore a very common epistatic mutant, has decreased hydrogen bond occupancy of the E134–R159 salt bridge from 64% to 28%. Note that there is ca. 25 Å between the location of residue 26 and the position of the salt bridge, clearly pointing to an allosteric communication (Figure 4c; salt bridge located at the back). In stark contrast, resistance conferring single mutants display significant shifts in the hydrogen bond occupancies.

A mutant we have previously studied in detail is L28R whereby we had shown substantially increased interactions between the substrate DHF and the enzyme, mainly utilizing the additional hydrogen bond donors/acceptors in the side chain replacement.^39,43^ In fact, based on these dynamical shifts at the binding site, we have recently proposed a modified inhibitor of DHFR that successfully distracts the evolutionary trajectories away from the detrimental L28R mutant.^10^ The new analysis also uncovers substantially increased occupancies of A107–R158 and R12–D127 residue pair interactions, stabilizing the β sheet in the loop domain (Figure 4e). L28R is also unique in that it is the only mutant for which the order parameter is reduced (local relaxations occur in a more unrestricted environment) compared to the WT protein.

Mutants in the W30 position are also often observed, and they have a similar effect on both *k*_cat_ and *K*_m_ (Table 1). Here, using a comparison of the effects at position 30 in Figure 4f,g, we find that this result is due to completely different underlying mechanisms. In our previous work, we were able to pin the effect of the more frequent W30R mutation to the formation of a salt bridge with E139 which indirectly releases the tension in the tight binding pocket while all other hydrogen bonds are occupied within 30% of the WT values.^43^ However, a similar mechanism was not traced for the W30G mutant. Our hydrogen bond analysis shows that, despite occurring in the same location, W30G induces a completely different effect on DHFR. Namely, a triad of dynamical hydrogen bonds between S3–E90–Y111 are substantially decreased while there is a direct stabilization of the substrate DHF by a nearly 50% increase in its hydrogen bonding with R57 (Figure 4f). Thus, the cavity formed by the shrinkage in the side chain volume at position 30 translates into a decrease in the support for maintaining the nearby triad S3–E90–Y111, and this new flexibility (increased entropy) is compensated as a strengthened interaction at the binding site.

We note that even though both W30G and L28R have increased interactions with DHF (by ∼40% and 45%, respectively; Table 2 and Figure 4e,f), both involve affecting hinge regions that were postulated to be the root cause of the differences in the activities of human and *E. coli* DHFR,^76^ albeit at two different positions. The analysis reveals that while W30G loses the S3–E90–Y111 interaction network near hinge 1 (residues 86–88) by substantially increasing the DHF–R57 interaction, L28R gains two additional hydrogen bonds between the M20–FG loops (R12–D127), and the A107–R158 between hinge 2 (residues 105–107) and the C-terminus. The hydrogen bond of A107–R158 in L28R may enhance a twisting motion of the ABD which would mimic an accepted mechanism for human DHFR. Additionally, the flexibility provided to G86 enables W30G to work against the translational/rotational entropy penalty. Nevertheless, unlike the other mutants and the WT, their *τ*_f_ distributions are widespread, spanning a 10 ps to 10 ns range which might be a result of their direct effect on the binding site interactions (Figure S5).

### Hydrogen Bond Dynamics Disclose a Cryptic Site along the CD Loop

**Figure 5:**
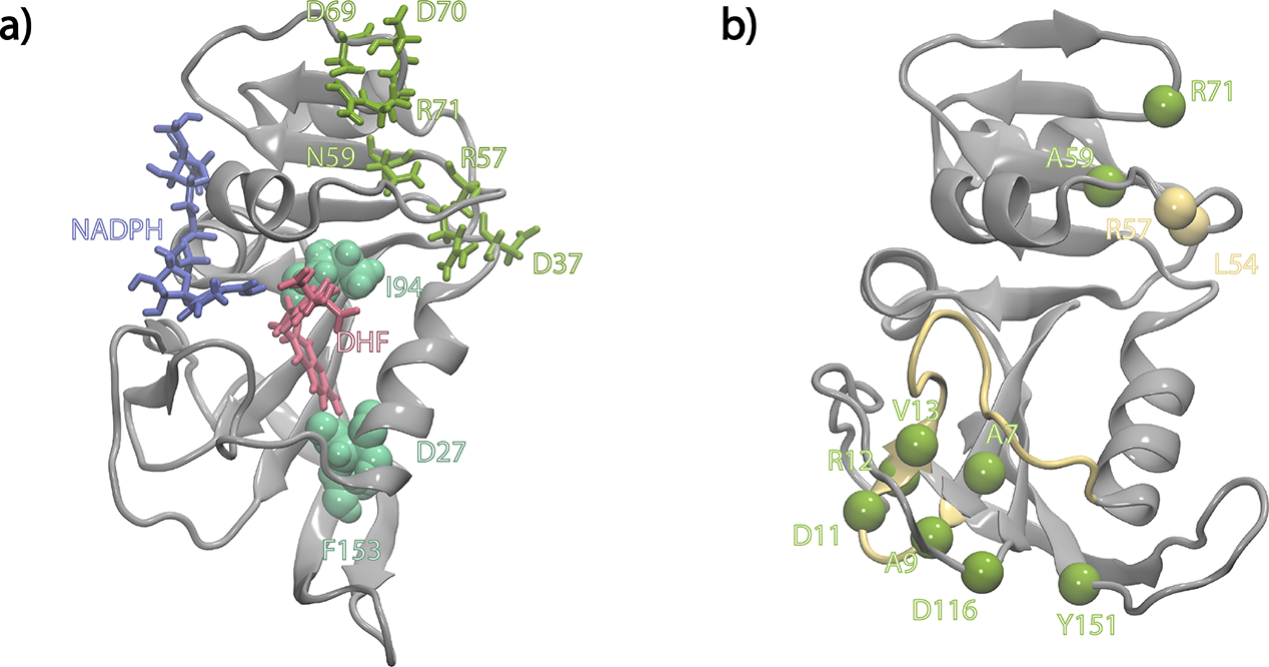
Stabilization of the cryptic site. (a) I94L, D27E, and F153S mutants (green) disrupt the hydrogen bond occupancies in the loop domain residues shown in olive. Positions of DHF and NADPH are shown to guide the eye. (b) Residues whose hydrogen bond occupancies are disrupted are displayed for the N59A mutant. Color coding is the same as in Figure 4.

Despite having previously disclosed some of the above-mentioned resistance mechanisms by closely monitoring the trajectories, systematically following the hydrogen bond occupancy changes has allowed disclosing a common mechanism that allosterically affects the binding cavity. D27E, I94L, and F153S mutants display a common, distinct hydrogen bond dynamics pattern whereby N59–R71 and R70–R71 bonds are ruptured, and the D69–R71 hydrogen bond is permanently formed (Figure 4d,h,j). Although this region also draws attention due to its low order parameters in NMR analyses,^67,77−80^ it has not previously been scrutinized in detail before. In Figure 5a, we display the locations of these three residues (green) as well as the residues involved in the cryptic site dynamics (olive). The former are in the loop domain of the enzyme, D27 and I94 being in direct contact with the ligand, while the latter are in the ABD. This long-range effect is not unusual in light of the fact that residues 67–69 on the CD loop are coupled in stability and function to G121 on the FG loop, despite separation exceeding 25 Å.^81^

In fact, previous studies derived from the NMR relaxation experiments show that the 67–69 region has low order parameters in the enzyme:folate:NADPH ternary complex.^67^ Upon M42W mutation, this region becomes flexible in the apo form, whereas methotrexate binding rigidifies it.^78,80^ Energetic coupling between the M20, the FG loop, and region 67–69 has also been demonstrated by ensemble-based computational modeling, and it was suggested that stabilization of the enzyme upon folate binding causes destabilization of the CD loop.^82^ In general, for the subset of advantageous mutations destabilizing the protein, it has been argued that mutations induce breathing motions, which in turn accelerate product release and is rate-limiting in the WT at neutral pH.^5^ Together with our findings, it may be argued that since residues 67–69 are affected by ligand binding, and their hydrogen bond dynamics greatly influence the flexibility of the ABD, the breathing motions of DHFR might be controlled by the presence/absence of the R71–N59 which in turn affect the rate of product release.

To see further if the interactions in this triad consistently affect catalytic activity, we have combed through the deep mutational scanning data on *E. coli* DHFR.^5^ We find that the activity of DHFR is quite sensitive to mutations at position 59; mutating N59 to small hydrophobics (PCGAVI) and D is beneficial, but replacing it with large hydrophobics (LMWFY), R, and Q is intolerant. To see if position 59 has an allosteric role, we have performed additional MD simulations on the N59A mutant using the same protocols. We find that this mutation promotes a cluster of hydrogen bonds at the loop domain, confirming the allosteric role of this region (Figure 5b). Interestingly, despite the lack of side chain donors/acceptors at position A59, the mutant maintains contact with the CD loop 23% of the time through a backbone hydrogen bond. The contact between R57 and DHF is permanently lost, as is a backbone hydrogen bond between L54 and N59. The region spanning residues 7–13 acquires a host of interactions for more than 50% of the time that were absent (A7–V13) or rare (A9–D11 and D11–R12) in the WT. Moreover, we find via Mole 2.0 cavity analysis^83^ that this allosteric region forms a pocket between residues 59 and 71 that may be used as a target for designed drugs.^84,85^

## Conclusions

In this study, we decipher the link between the nanosecond scale side-chain dynamics of the enzyme and function related relaxations of its ligand at the hydride transfer bond (γ). This discovery enables us to determine the underlying working mechanisms of trimethoprim-resistant DHFR mutants,^86,43^ by tracing large changes in hydrogen bond occupancies. We identify distinct modes of action: (i) Some mutations directly work on the DHF binding cavity. (ii) In a single case (W30R) a salt bridge established outside the binding cavity affects the fate of enzyme kinetics. (iii) Some mutants enable an interesting shift in the hydrogen bond network at the distant CD loop. D27E displays a combination of modes i and iii. Other mutations which do not have any significant changes in their hydrogen bond occupancies are also the ones seldom observed as the first mutation, but rather appear later in evolutionary trajectories and are therefore epistatic (I5F, A26T, R98P).

Perhaps the most striking result of our hydrogen bond occupancy analysis has been to find that seemingly three unrelated mutations that are frequently observed as first replacements in the morbidostat, i.e., D27E, I94L, and F153S, all have a common allosteric effect (Figure 4d,h,j). These mutants display a distinct hydrogen bond pattern whereby N59–R71 and D70–R71 interactions which occur intermittently in the WT trajectories are permanently ruptured, and the D69–R71 hydrogen bond is completely stabilized. However, it is necessary to note that these mutants also maintain a relative flexibility of the CD loop within their structure. Another observation is that the flexibility of the FG loop alone does not provide the required fitness. Resistance conferring mutants always include FG loop flexibility with CD loop flexibility, indicating an allosteric coupling.

Using kinetics experiments on double mutants of M42 and G121 at distal positions to the active site, the coupling between these two residues residing on opposite domains was unequivocally shown.^87^ However, the physical nature of the process remained elusive. Here we propose the tuning of the motions of the entire enzyme to the substrate as a possible explanation, rather than it being due to a specific network of interactions connected across the enzyme. In fact, the vicinal layer of solvent might well be playing the role of conduits of the changes observed. Therefore, the concept of energy–entropy compensation becomes vital in the formation of the ligand–enzyme complex. When the binding process becomes tighter with a sizable enthalpic contribution, the motions of both enzyme and ligand become restricted, resulting in much lower entropy. Moreover, the enthalpic contribution must overcome the translational/rotational entropy penalty for favorable binding,^88^ which may be achieved by residual motions until the binding strength allows this to happen.^89^

While the whole protein is involved in fine-tuning to the effects of point mutations, we can nevertheless use the information we have obtained to propose strategies to target antibiotic resistance. Knowledge of the specific mechanisms of the mutations might lead to the design of inhibitors that stir the evolutionary trajectories to carry less benign mutants, i.e., those where the original drug still displays activity. As a new strategy, one may directly target R57 due to its enhanced interactions with the substrate in the W30G mutant and its indirect involvement with activity through formation of a salt bridge with D37 in the I94L variant. We rely on these dynamically formed hydrogen bonds as a valid strategy, since we have already successfully taken advantage of the interactions of the R28 side chain in our proof-of-concept study whereby the 4′DTMP derivative impedes the emergence of this variant.^10^ A novel design approach that has also emerged from this study might be to utilize the cryptic site aligning the CD loop which we will pursue in future work.

## Supporting Information Available

Frequency distribution of changes in hydrogen bond occupancies, RMSD profiles in MD simulations, relaxation profiles of DHFR binding site residues, *ω* bond relaxations, residue specific relaxation profiles of side-chains, and common hydrogen bonds lost/gained in resistance conferring mutants (PDF)

## Notes

*Data and Software Availability*. All MD trajectories are stored on a local server and will be shared upon request due to the large file sizes. MATLAB R2019a codes calculating methyl side chain correlation functions and parameter fits are available at https://github.com/midstlab/MethylRelaxationCurvesandAssociatedFits. Python 3.7 codes calculating the hydrogen bond occupancy shifts are available at https://github.com/midstlab/HydrogenBondAnalysis.
