# Supplementary material for: DHFR Mutants Modulate Their Synchronized Dynamics with the Substrate by Shifting Hydrogen Bond Occupancies
Source: J Chem Inf Model. 2022 Aug 19;62(24):6715–26. doi: 10.1021/acs.jcim.2c00507 (PMC9795552; doi:10.1021/acs.jcim.2c00507)
Supplement: Supplementary file 1 — ci2c00507_si_001.pdf [file ci2c00507_si_001.pdf]

Supplementary Information for

DHFR mutants modulate their synchronized dynamics with the substrate by shifting hydrogen bond occupancies

Ebru Cetin, Ali Rana Atilgan, Canan Atilgan*

Faculty of Engineering and Natural Sciences, Sabanci University, 34956, Istanbul, Turkey.

*canan@sabanciuniv.edu

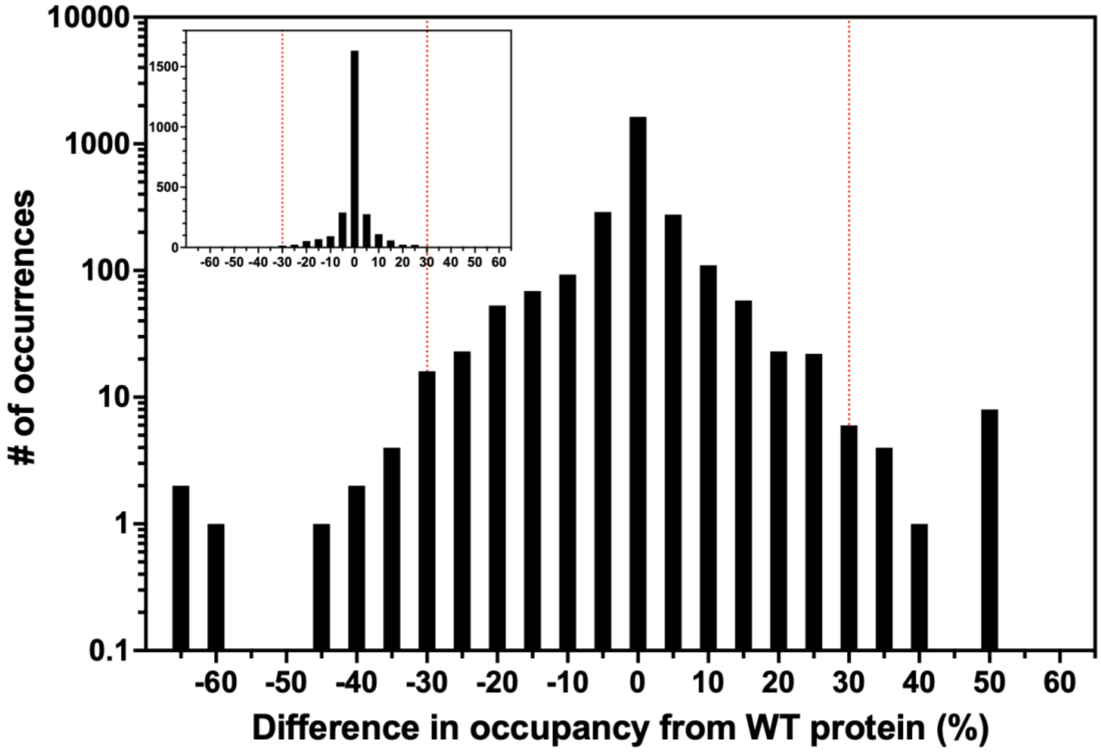

Figure S1. Frequency distribution of the changes in hydrogen bond occupancies relative to WT for all mutants (inset: same data on normal scale along the *y*-axis).

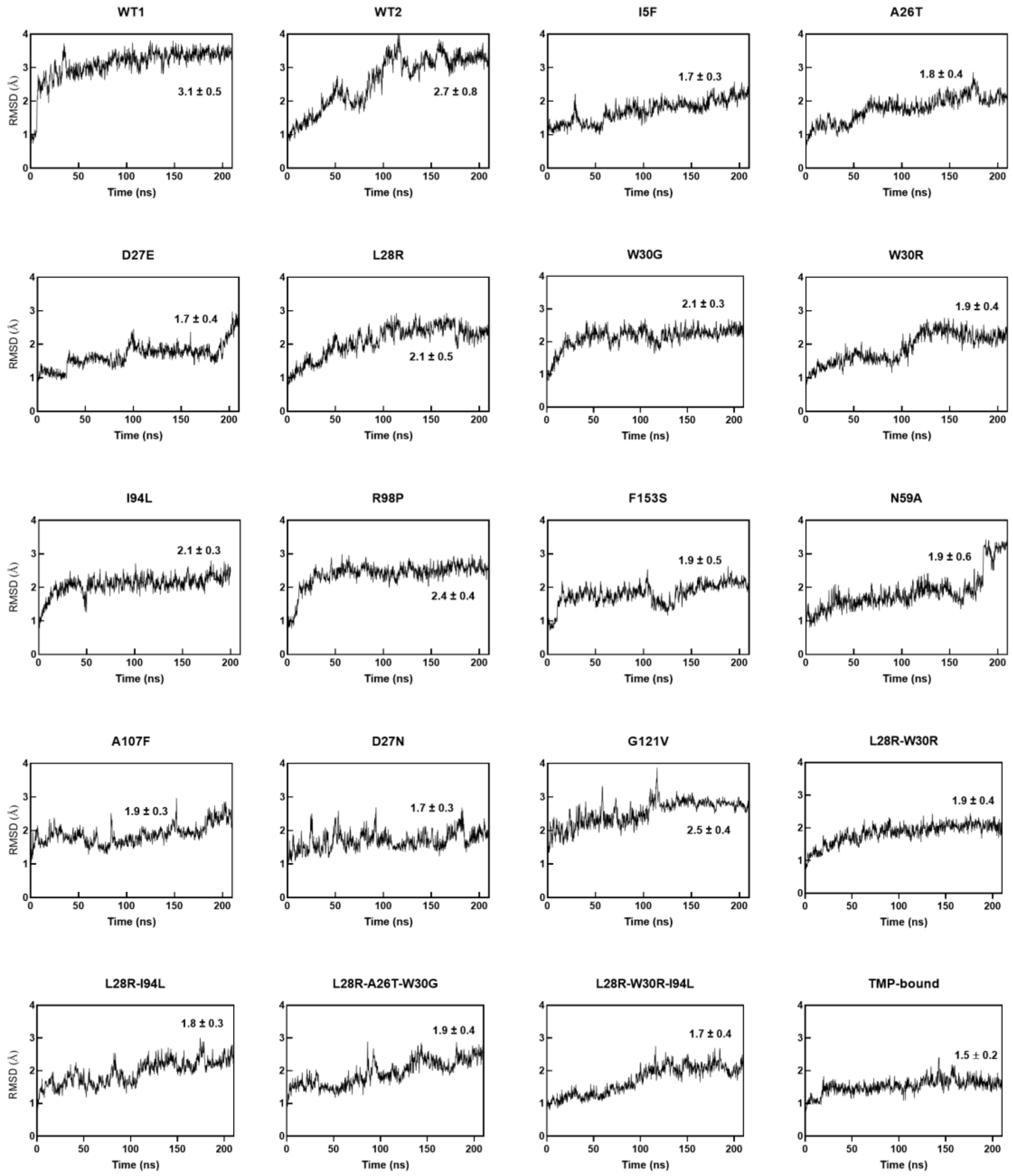

Figure S2. Root-mean-square deviation (RMSD) profiles of backbone atoms with respect to the minimized structure in the 210 ns trajectories for each system studied in this work.

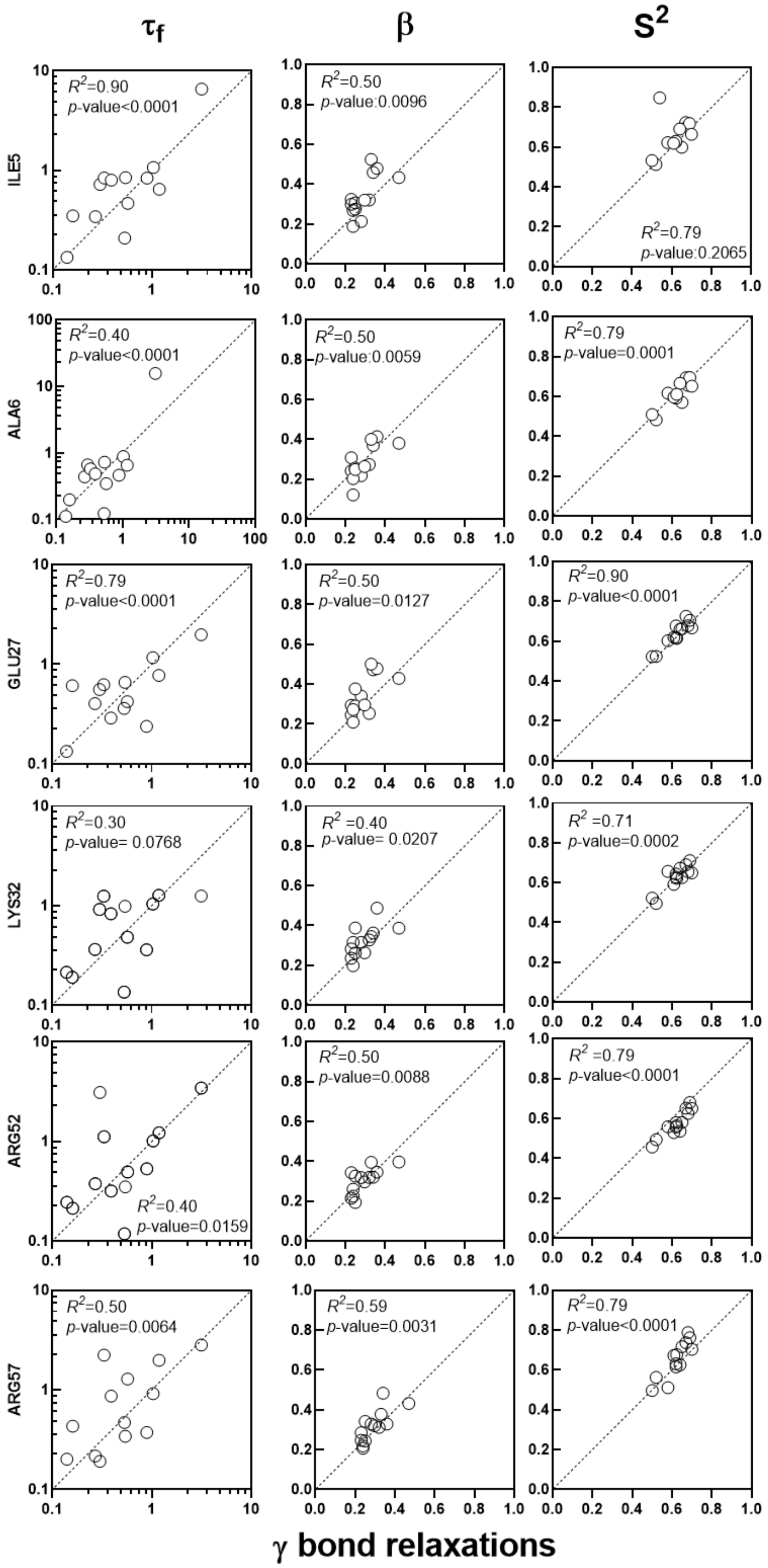

Figure S3. Relaxation profiles of DHFR binding site residues for all single mutants.

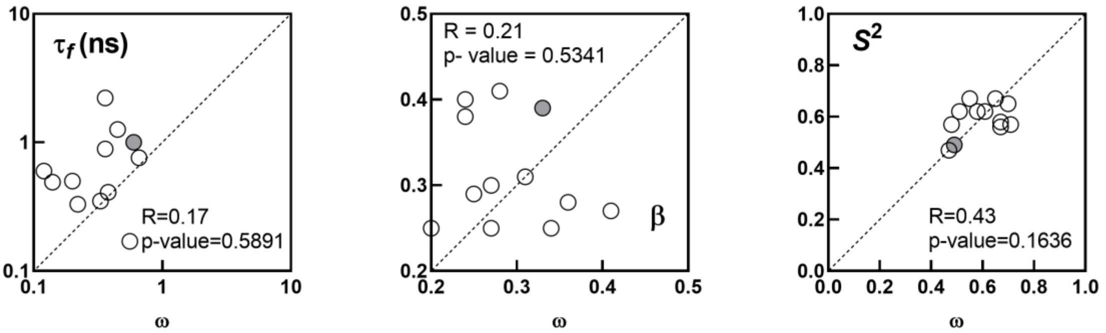

Figure S4. Comparison of enzyme mean (over 159 residues) versus that of the ω bond adjacent to the γ bond (see figure 1a) relaxations for all the systems studies. WT is shown by the gray filled circle. y=x line shown to guide the eye in each case.

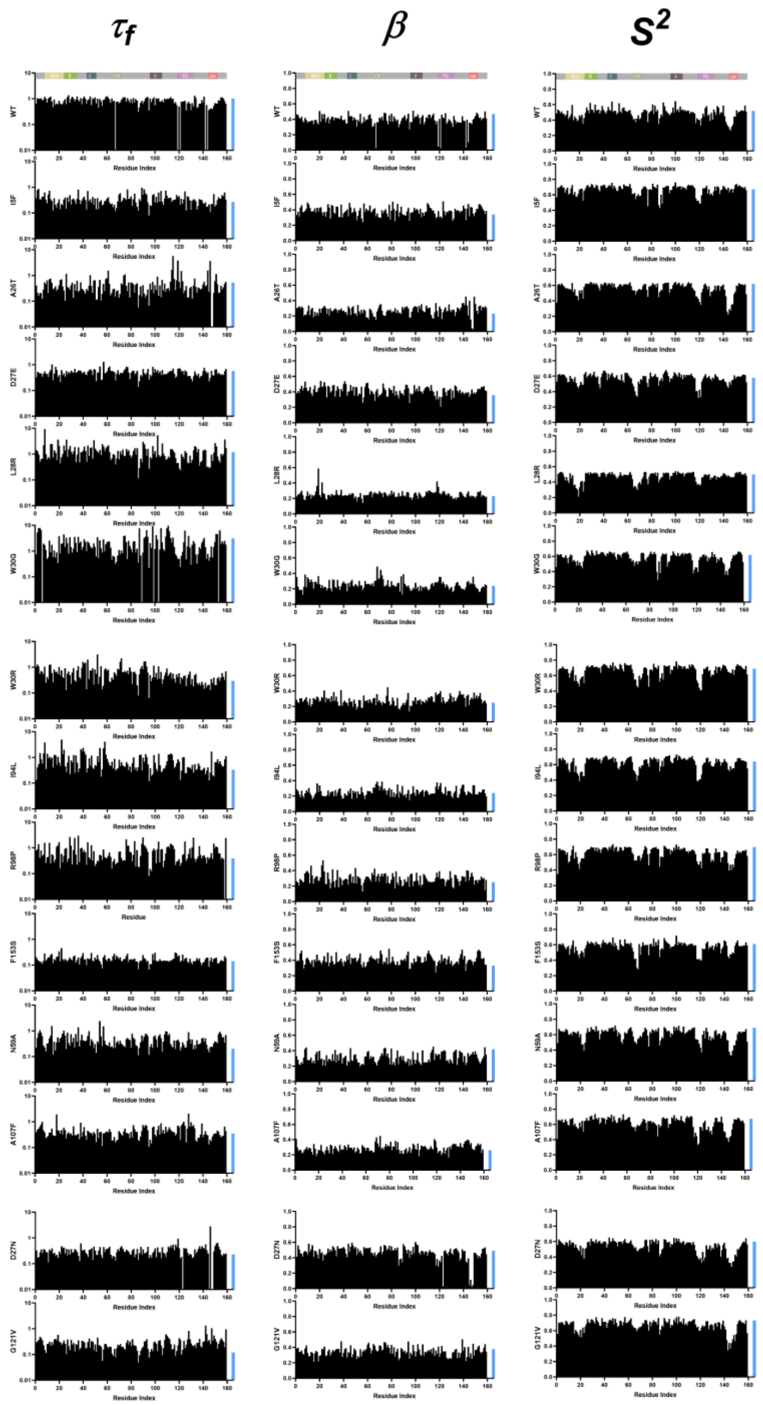

Figure S5. Individual relaxation profiles of C_α-C_β bonds (black) and the respective γ bond relaxations (blue) for all mutants. Their averages are displayed in Figure 3.

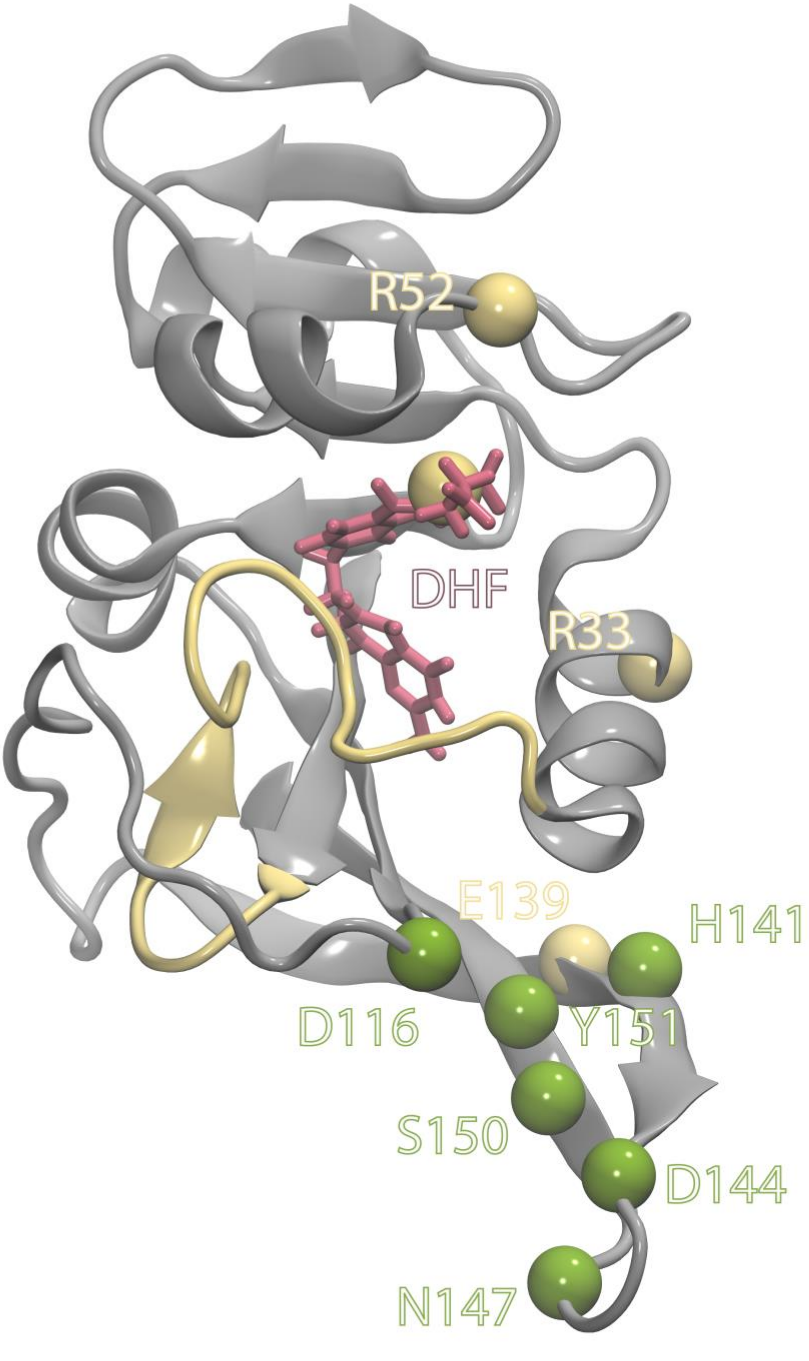

Figure S6. Common hydrogen bonds lost (yellow)/gained (green) in resistance conferring mutants.
